# Supplementary material for: Tunable colloid trajectories in nematic liquid crystals near wavy walls
Source: Nat Commun. 2018 Sep 21;9:3841. doi: 10.1038/s41467-018-06054-y (PMC6155032; doi:10.1038/s41467-018-06054-y)
Supplement: Supplementary file 1 — Supplementary Information [file 41467_2018_6054_MOESM1_ESM.pdf]

Tunable colloid trajectories in nematic liquid crystals near wavy walls

Supplementary Materials

Luo *et al.*

Supplementary Figures

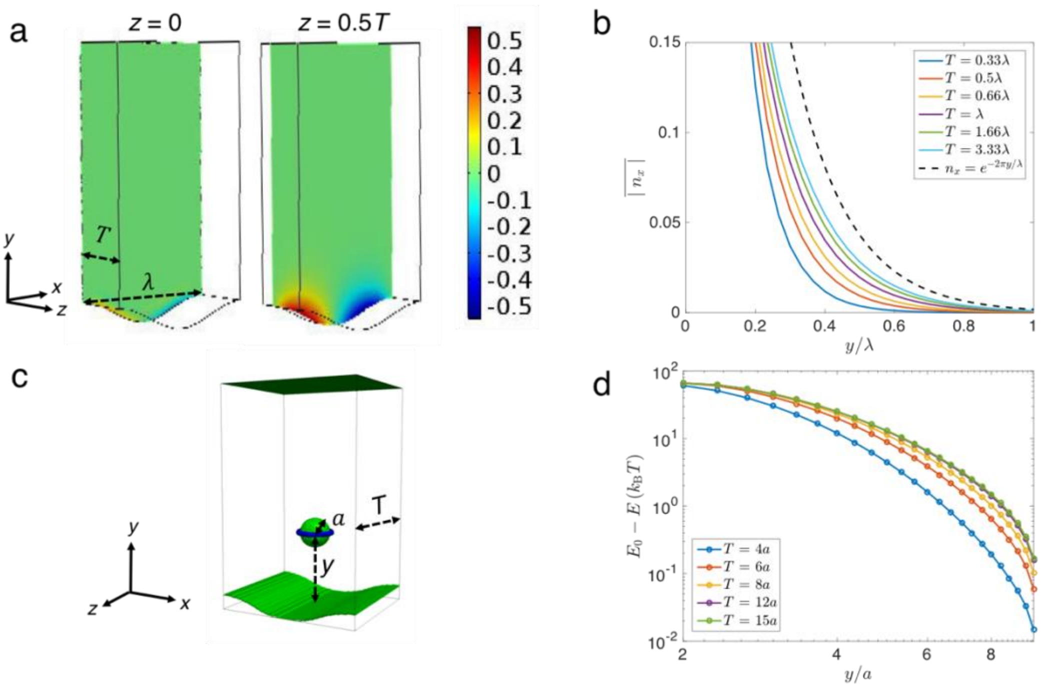

Supplementary Figure 1. Confinement truncates the decay length of wall perturbations in the director field. (a) The color denotes the deviation from 0 of the n_x component of the director field, which decays with distance *y* from the wavy wall. The color bar denotes the magnitude of the distortion. At $z = 0$, the wall imposes uniform planar anchoring; thus, at this slice, $n_x = 0$. At the plane $z = 0.5T$ corresponding to the midplane between top and bottom plates, the distortion is strongest. (b) The absolute value for n_x, averaged across the gap, versus the colloid's distance *y* from the bottom of the well. The dotted line corresponds to exponential decay, the limit for an infinite gap width. (c) Schematic of the configuration for LdG simulation of a colloid of radius *a* with center of mass (COM) at height *y* above the bottom of the well in a cell of thickness *T* with a Saturn ring defect. (d) The energies from LdG numerical modeling for cell of thickness *T* vs. *y*, normalized by radius of the colloid.

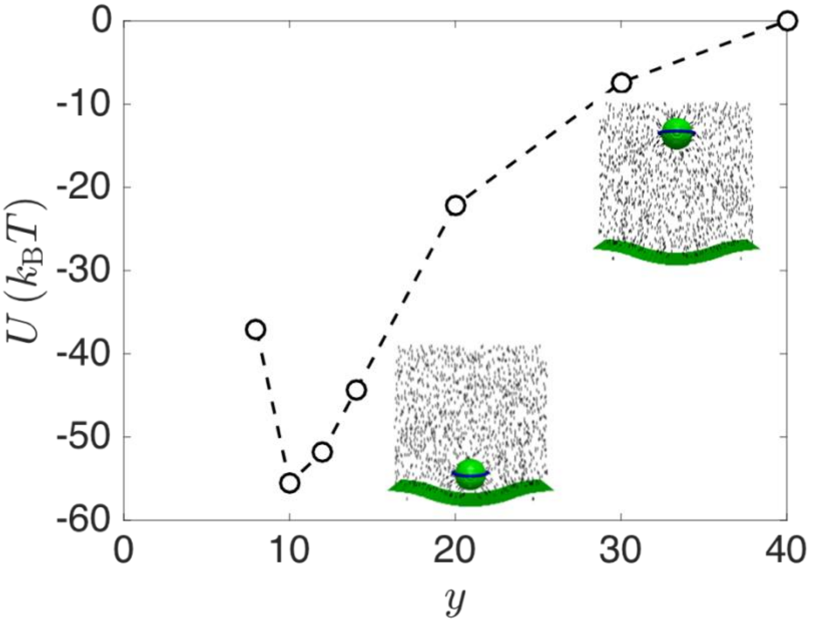

Supplementary Figure 2. Potential energy by Landau de Gennes (LdG) simulation. The energy of a colloid with Saturn ring defect is simulated by placing it at different distances above a well with normalized radius R/a=7, and normalized wavelength λ/a=8.5, where a is the radius of the colloid.

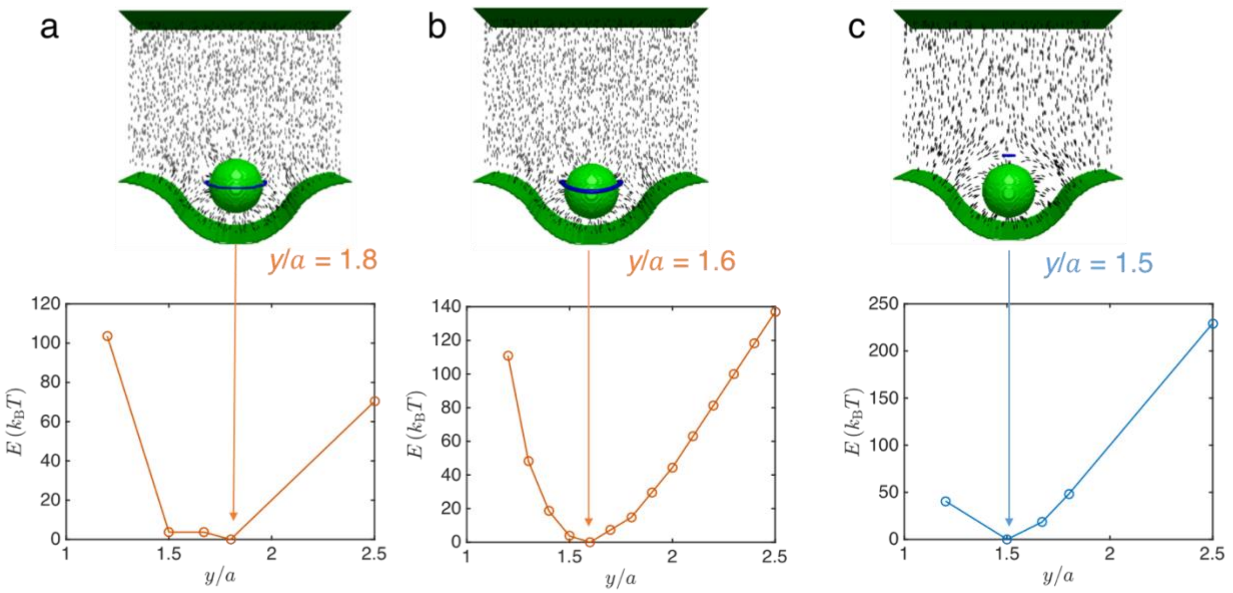

Supplementary Figure 3. Energy of the quadrupole and dipole in wells. (a) Saturn ring reaches lowest energy at *y/a* = 1.8 (splay matching) when simulated with 2.6x core energy where the ring does not distort. (b) Saturn ring reaches lowest energy at *y/a* = 1.6 (distorted Saturn ring) when simulated with 1x core energy. (c) Dipole reaches lowest energy at *y/a* = 1.5 when simulated with 2.6x core energy and initialized with dipolar configuration around the particle. *a* = 135 nm, *R* = 243 nm, *R/a* = 1.8 in all cases. We have verified that the same equilibrium position hold for system of different sizes as long as the core energy is the same as the cases simulated here. The reference energy is set to be the minimum energy stage corresponding to the equilibrium position.

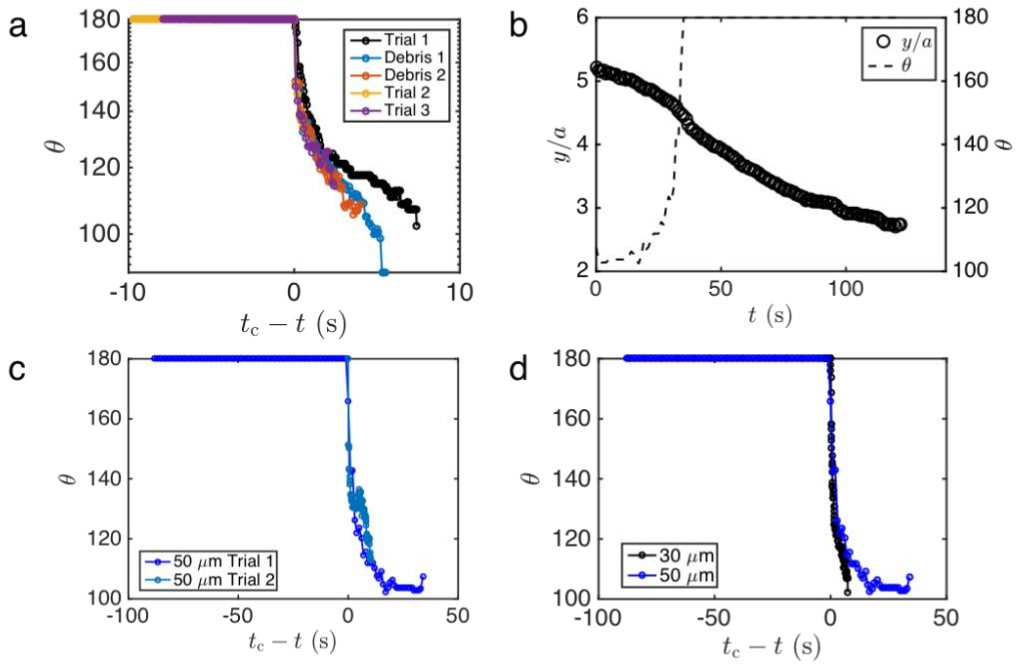

Supplementary Figure 4. Quadrupole to dipole transition dynamics. (a) Same as Fig. 4d but including two cases which include debris. The angle evolution is highly reproducible in all cases near the transition time t_c. (b) Tracking center of mass (COM) height and polar angle θ evolution during the quadrupole to dipole transition for well of 2*A* = 25 μm. (c) Two instances of quadrupole-to-dipole transition in well 2*A* = 25 μm with reproducible dynamics. (d) Defect dynamics of two transitions which happened near walls with 2*A* = 15 μm and 2*A* = 25 μm wells respectively show that, even if the systems are different, the dynamics of the transition is similar. The legends in a, c, d denote video number of the trajectories tracked.

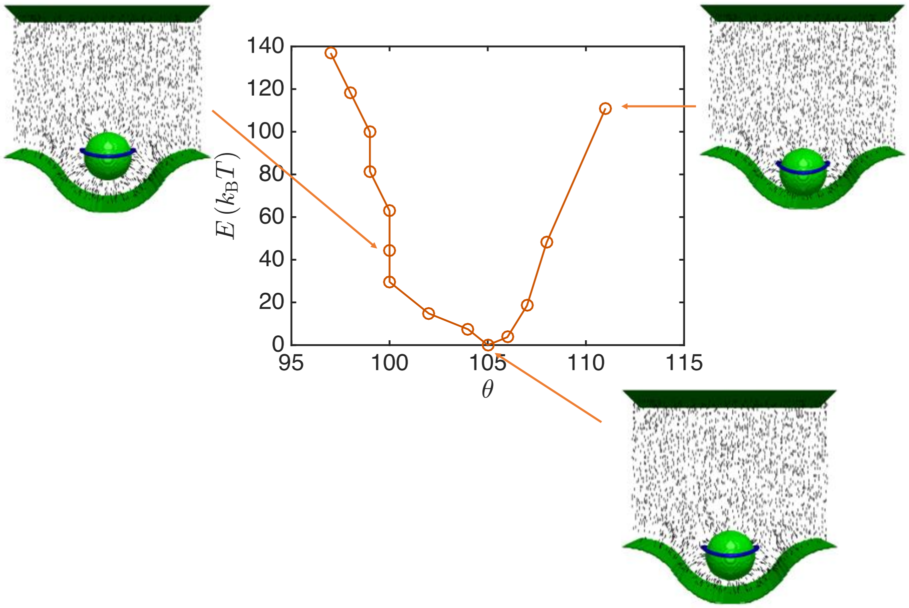

Supplementary Figure 5. Simulation of total energy of the system *E* versus the reaction coordinate *θ*. The reaction coordinate denotes the polar angle of the Saturn ring. Snapshot of the equilibrium simulation is attached where the particle is located at various distances from the well. The particle has minimum energy when it is located at the equilibrium position. The reference energy is that of particle at equilibrium position.

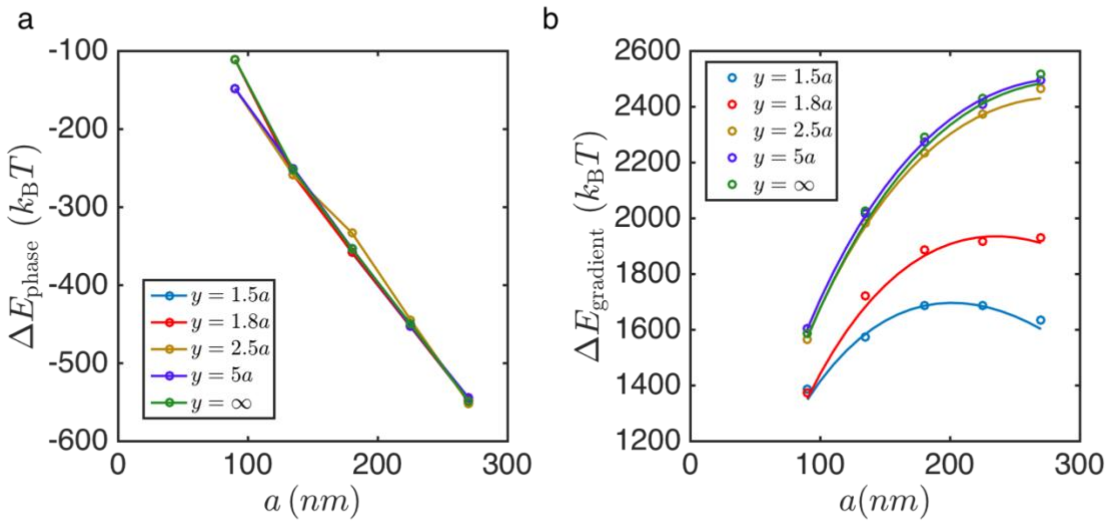

Supplementary Figure 6. Simulation energy scaling. The energy of the simulation consists of two parts, the phase free energy ($\Delta E_{\mathrm{phase}}$, associated with the defect) and the difference in gradient free energy ($\Delta E_{\mathrm{gradient}}$, associated with the distortion of the field). (a) The difference between the dipole and Saturn ring in phase energy scales linearly in a. This scaling emerges because the defect energy of the dipole does not grow while that of the Saturn ring defect does grow with the linear dimension of the system. (b) The difference in gradient free energy has a linear part (~a) and a logarithmic part (~a log a) based on [4]. Here it is fitted to form $k_1 a + k_2\ a\ \log a + k_3$. At large radius, this is linear in a.

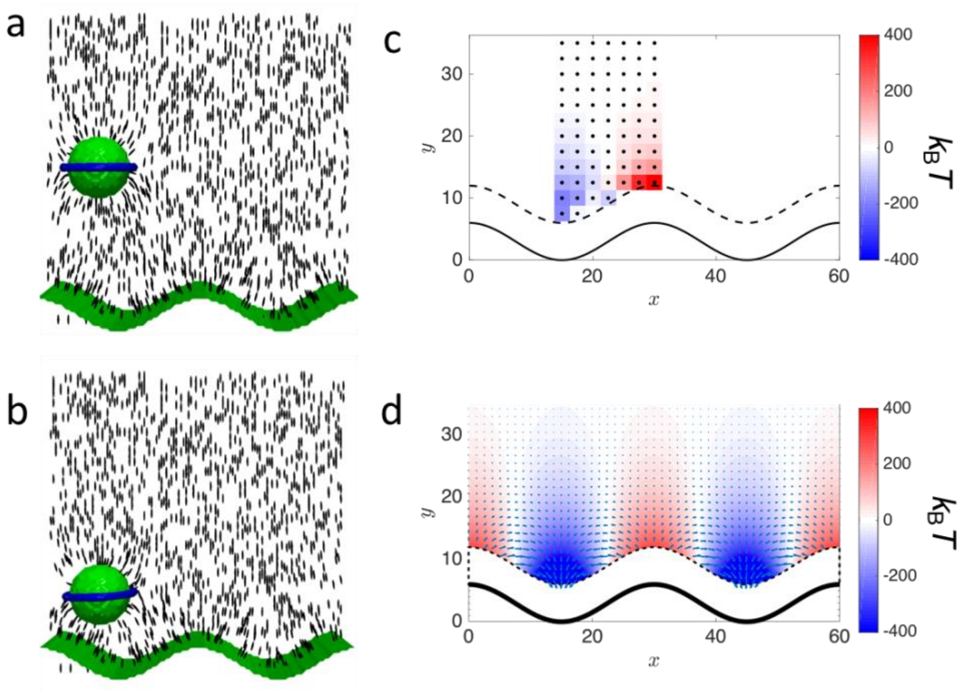

Supplementary Figure 7. Mapping the energy landscape by sampling and calculating particle trajectories by force field. (a, b) Sample LdG numerical modeling results with a colloid centered at two different grid locations above a wavy wall. (c) Discretized form of energy by choosing the site above the center of the well ($\lambda/2$, λ) as the reference energy. (d) Smoothed energy field extended by assuming symmetry and periodicity.

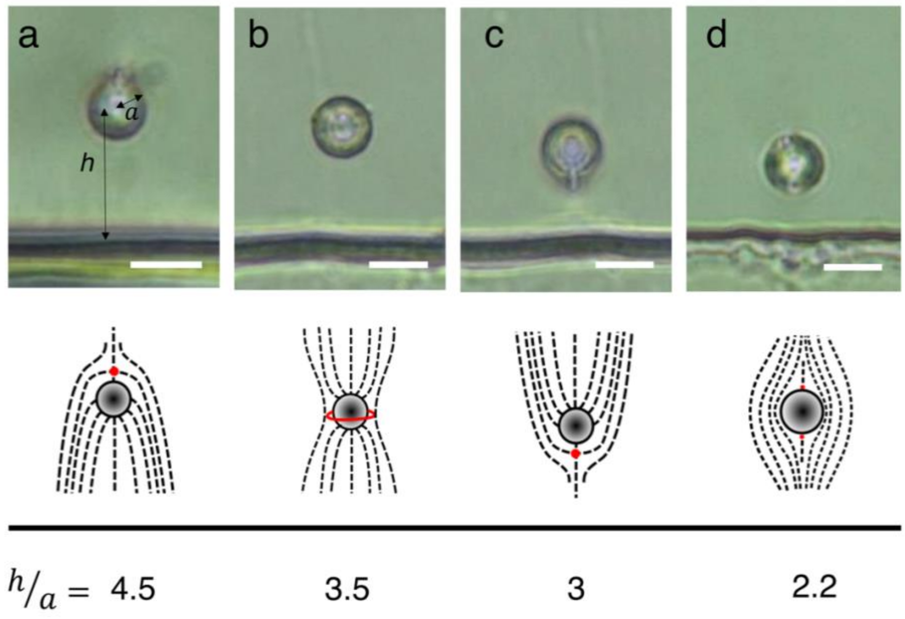

Supplementary Figure 8. Equilibrium distance of particles away from a planar homeotropic wall. Particle diameter $2a$ = 8.7, 10, 10.2, 9.8 μm in a-d. The final equilibrium particle position away from a flat wall for (a) a dipole with its point defect oriented upwards, (b) a colloid with Saturn ring defect, (c) a dipole with its point defect oriented downwards, (d) a colloid with boojums. Defects are illustrated schematically in red in all cases. The scale bars are 10 μm.

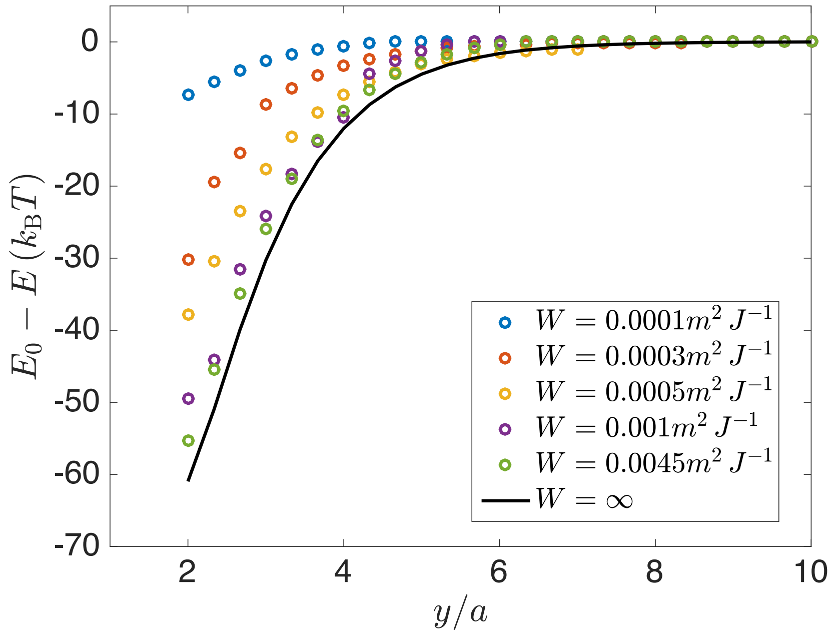

Supplementary Figure 9. Effect of anchoring. The energy of a colloid with Saturn ring defect is simulated by placing it at different distances above a well in a cell of $T = 4a$. For realistic anchoring strength, the energy profile as the particle position changes near the wall remains very similar to the case of infinite anchoring (solid line). If we decrease the anchoring by 10-fold, binding energy (the energy difference between when the particle is far and when it is near the well) decreases, and so does the gradient. Therefore, we also expect the range of interaction to decrease.

Supplementary Notes

Supplementary Note 1: Effects of confinement. We study the motion of colloids confined in a sandwich cell near a wavy wall. In a related study, absent wavy wall, the effect of confinement on inter-particle potential for particles in a sandwich cell has previously been explored in [1]. This study showed that the elasticity-mediated potential for particle pair interactions decreased sharply for center to center particle separations that were large compared to the thickness of the cell. In our sandwich cell, oriented planar anchoring is enforced in the *y*-direction on the two glass coverslips, in the plane of observation. To investigate the effect of this anchoring on the orientation of the director field in the bulk, we use COMSOL and LdG to investigate the director field for a series of cell thicknesses.

COMSOL is used to calculate the average deviation of the director field above the well with distance *y*. The nematic is assumed uniaxial, with equal Frank elastic constants. The procedure, detailed in the Methods section, is briefly restated here. For a given geometry constructed in COMSOL, we solve for components of the director field. The components are governed by Laplacian $\nabla^2 n_i = 0$ where i = x, z, and $n_y = \sqrt{1-n_x^2-n_z^2}$. Furthermore, the following boundary conditions are imposed: Periodic boundary conditions are enforced in *x*, uniform planar anchoring ($n_x|_{\text{glass}} = 0,\ n_z|_{\text{glass}} = 0$) is enforced on the top and bottom glass walls and perpendicular anchoring ($n_x|_{\text{wavy wall}} = \dfrac{-F'(x)}{\sqrt{1+\left(F'(x)\right)^2}}$, $_{\text{wavy wall}} = 0$) is enforced on the wavy wall; $y = F(x)$ defines the shape of the wavy wall. At the boundary opposite the wavy wall at the top of the simulation cell, a free boundary condition is applied. In the single constant approximation, these boundary conditions require that $n_z = 0$ everywhere. In a system with no colloids, the distortion is determined by variation of n_x alone. At the glass slide, (Supplementary Figure 1a, left panel) the oriented planar anchoring is enforced. In the bulk, the wavy wall is a source of distortions (Supplementary Figure 1a, right panel). We have performed these simulations for a number of cell thicknesses *T* for fixed λ = 30; results for *T* = 15 mesh spacing is shown in Supplementary Figure 1a. By considering slices in *xz*-plane (constant *y*), we determine the average absolute values of n_x in that plane. This quantity captures the magnitude of the distortion; a value close to 0 means the director field is

oriented the same way as the far-field director. As expected, the distortion decays with distance from the wavy wall (Supplementary Figure 1b). For infinite T, the director distortion decays exponentially with decay length λ; this asymptote is plotted as the dashed line in Fig. Supplementary Figure 1b. For finite T, the distortion field decays more rapidly, owing to the anchoring energies on the top and bottom of the cell.

To relate these findings to the distance H^* at which the colloid interacts with the wall, LdG simulation is used to calculate the total energy of the system with a colloid with homeotropic anchoring in a Saturn ring configuration. The colloid of radius a is placed at various distances y from the wavy wall, centered above the well (Supplementary Figure 1c). The energy of the system is plotted against the distance y normalized by the radius a on a log-log plot for different cell thicknesses T (= 4a, 6a, 8a, 12a, 15a, Supplementary Figure 1d). The plot confirms that confinement diminishes the range of the interaction of the wall.

Supplementary Note 2: Energetics of quadrupole-to-dipole transition. To gain insight into the energetics of the quadrupole to dipole transition, we use a combination of numerics and theory. LdG numerical modeling is used to compare the energy of colloids with dipolar and Saturn ring configurations at different distances y away from the wavy wall. In our study, we increase the nematic defect core energy by 2.6x to accommodate for the small size of our simulation compared to experimental scales for all simulation of dipoles and those for Saturn rings used to compare to the results of dipoles. We do this in order to stabilize a dipolar configuration in the numerics for colloid radii as small as 90 nm, allowing us to model both the colloid and the larger wavy wall in a box of reasonable size. The Landau-de Gennes free energy stabilizes the nematic phase over the isotropic phase with a free energy density f_{phase}:

$$f_{phase} = \tfrac{1}{2} A \cdot Tr(Q^2) + \tfrac{1}{3} B \cdot Tr(Q^3) + \tfrac{1}{4} C \cdot Tr((Q^2))^2 \qquad (4)$$

To increase the defect core energy, we replace the ratios $B/A \approx 12.33$, $C/A \approx -10.06$, commonly used to model 5CB [2], with $B/A = 106$, $C/A = -86.5$. With $A < 0$ held fixed, this replacement increases by a factor of 2.6 the magnitude of the minimum value of f_{phase}, which sets the defect core energy density.

To simulate the dipolar configuration rather than the Saturn ring, we initialized the system with a dipole-like director field. The spherical colloids are placed near the wall at various heights *y*, with two independent trials for each colloid position, one with a dipole resulting from the dipole-like initialization, and one with a Saturn ring resulting from a uniform director field initialization. The colloid with the Saturn ring defect achieves minimum energy at $y = 1.8a$, corresponding to the splay-matching height (Supplementary Figure 3a). The colloid with the dipolar configuration sits lower inside the well, achieving its minimum energy at $y = 1.5a$ (Supplementary Figure 3c). In both cases, the lowest energy is chosen as the reference energy. In comparing the energy of the Saturn ring and the dipole, we must keep in mind that the Saturn ring is more stable for very small colloids, while the dipolar configuration is stable for larger colloids ($a > 720$ nm, [3]). Thus, the Saturn ring always has the lower energy in the numerics, while the opposite is true in experiment.

Supplementary Note 3: Generating the energy field and sample trajectories in Fig. 7a. In order to gain insight into the energy landscape of a colloid near the wall, Landau-de Gennes (LdG) numerical modeling was performed for a colloid located at different locations between two identical wells. The colloid center of mass was placed at a fixed location. The corresponding energy field for the NLC was found. The colloid location was then moved to a new location on a grid. The spacing between COM locations was set to 2.5 times the simulation mesh size, corresponding to 11 nm. The rest of the simulation parameters are as follows, with lengths given in multiples of the simulation mesh size: Colloid radius $a = 12$, corresponding to 54 nm; wavelength of the well $\lambda = 60$, corresponding to 270 nm; amplitude $A = 6$, corresponding to 27 nm, with well curvature radius R of roughly 22, corresponding to 98 nm. Thus, $\lambda/a = 5$ and $R/a = 8$ and adjacent grid points are separated by $\sim 0.2a$. The colloid surface imposes homeotropic anchoring of infinite strength.

To calculate the liquid crystal free energy, the colloid is centered at a specified location on the grid and the Landau-de Gennes free energy is minimized numerically over the Q-tensor field; the details of the simulation can be found in the Methods section in the main text. The output from two representative colloid locations are shown in Supplementary Figure 7a, b. The discretized liquid crystal free energy as a function of colloid center position (denoted by black dots) is expressed in

the heat map shown in Supplementary Figure 7c, by choosing the energy when a colloid is located at (x, y) = (λ/2, λ) as reference, and performing the conversion from simulation units to real units (1 s.u. = 3.7 $k_B T$). Assuming symmetry and periodicity, we can fit a smooth function $g(x, y)$ over the entire space. By taking the gradient of this energy field $g(x, y)$, we obtain the direction of the elastic force field experienced by the particle. This elastic force includes the distortion and defect sourced by a colloid in the domain. Hills are repulsive, and wells are attractive in the case of a colloid with homeotropic anchoring in a Saturn ring configuration (Supplementary Figure 7d).

Finally, we simulate trajectories of a colloid released at different locations near the wavy wall. Consider a colloid placed at an initial position (x_0, y_0). The force balance on the colloid is simplified in the limits explored in experiment. Given the low Reynolds number applicable to the colloid's motion, acceleration is negligible. Furthermore, since the colloids move in a deterministic manner, Brownian motion is also negligible. Thus, the colloid moves quasi-statically along a path determined by the elastic force, which is balanced by viscous drag on the particle. We are interested here in the particle path, which is determined by the elastic force. The direction and magnitude of the elastic force at the initial location is calculated by the gradient of the field $g(x, y)$ at that point, as described above. Colloidal paths are simulated by updating the particle position by a step path length Δs, according to explicit Euler scheme:

$$\begin{cases} x_{i+1} = x_i - \frac{\partial g}{\partial x}\Big|_{x_i, y_i} \Delta s \\ y_{i+1} = y_i - \frac{\partial g}{\partial y}\Big|_{x_i, y_i} \Delta s \end{cases} \tag{3}$$

Trajectories starting from arbitrary locations can thereby be calculated, in qualitative agreement with experimental trajectories. We show that the trajectories are very sensitive to initial locations. This method does not take into account hydrodynamic interactions with the wall or Brownian motion.

Supplementary Note 4: Repulsion from a planar wall. In order to determine how colloids interact with a planar boundary, we place ferromagnetic colloids (2a = 8.7, 10, 10.2, 9.8 μm in Fig. Supplementary Figure 8a-d) with different defect configurations adjacent to a planar wall with homeotropic anchoring (Supplementary Figure 8). Upon releasing the magnetic field, the colloids,

repelled, migrate away from the wall. We track their trajectories and record their equilibrium location $y = h$. To confirm that the particles have reached their equilibrium positions, we track the particles for an additional 500 s to ensure that they do not move further from the wall. Since we are looking at the sample from a bird's eye view, the particles do not move under the influence of gravity. Particles reach different equilibrium heights based on the type and orientation of their defects. In particular, an oriented dipole with the defect away from the wall (Supplementary Figure 8a) has the longest range of repulsion from the wall to a distance of $h/a = 4.5$ while a colloid with boojums equilibrates merely $h/a = 2.2$ away from the wall. Furthermore, although the symmetry of these colloids can be approximated as multipoles in the far field, in the near field the details matter. Specifically, the director field between the upward-orienting dipole and the Saturn ring are similar (diverging splay), and that of the downward-orienting dipole and the boojum are similar (converging splay). As a result, these colloids behave similarly based on their near-field director configurations.

Supplementary References

[1] M. Vilfan, *et al.*, Confinement effect on interparticle potential in nematic colloids, *Phys. Rev. Lett.* **101**, 237801 (2008).

[2] M. Ravnik and S. Žumer, Landau-de Gennes modelling of nematic liquid crystal colloids, *Liq. Crys.* **36**, 1201–1214 (2009).

[3] H. Stark, Director field configurations around a spherical particle in a nematic liquid crystal, *Eur. Phys. J. B* 10, 311-321 (1999).

[4] H. Stark, Physics of colloidal dispersions in nematic liquid crystals, *Phys. Rep.* 351, 387–474 (2001).
