# Supplementary material for: Tunable colloid trajectories in nematic liquid crystals near wavy walls
Source: Nat Commun. 2018 Sep 21;9:3841. doi: 10.1038/s41467-018-06054-y (PMC6155032; doi:10.1038/s41467-018-06054-y)
Supplement: Supplementary file 3 — Description of Additional Supplementary Files [file 41467_2018_6054_MOESM3_ESM.pdf]

Description of Additional Supplementary Files

Supplementary Movie 1: Quadrupole to dipole transition. A Saturn ring transforms to a dipole at a metastable position remotely from the wall defined by the elastic energy field. This video is 1/3x slower than real time.

Supplementary Movie 2: Irreversible transformation. To eliminate surface pinning, an aqueous droplet, stabilized by Span 80®, is suspended in NLC. The inner phase is crosslinked and contains iron oxide. At $t = 102$ s, a magnetic field is applied to steer the colloid away from the wall. The transition is irreversible. This video is 10x faster than real time.

Supplementary Movie 3-5: Multiple trajectories in complex energy landscape. Upon releasing a homeotropic particle with Saturn ring defect between two wells, the particle took one of the three possible trajectories: Movie 3: left, Movie 4: right, and Movie 5: repelled upwards. These videos are 20x faster than real time.

Supplementary Movie 6: Colloid with a Saturn ring shuttling from one wall to the other. Between two walls whose waves are "in phase" a colloid with Saturn ring is repelled from the hill and lands in the wells on the opposite side. This video is 20x faster than real time.

Supplementary Movie 7: Dipole shuttling from one wall to the other. Between two walls whose waves are "out of phase", an upward orienting dipole is repelled from the hill and lands on top of the hill on the opposite side. This video is 20x faster than real time.

Supplementary Movie 8-9: Bistable dipoles between two wavy walls. Between two walls that are "in phase", a downward orienting dipole can dock either on the hill in one wall or inside the well on the opposite side. These videos are 3x faster than real time.

Supplementary Movie 10: Uniform well migration with gravity docking. A colloid with Saturn ring ($2a = 13$ μm) defect, moving under the action of gravity (tilt angle $\alpha = 11.2°$) docks inside the nearest

Supplementary Movie 11: Uniform well migration with gravity skipping. A colloid with Saturn ring defect ($2a = 13$ μm), moving under the action of gravity (tilt angle $\alpha = 12.3°$), follows an oscillatory path without docking. The period of the wells is uniform ($\lambda = 70$ μm). The video is 120x faster than real time.

Supplementary Movie 12: Goldilocks with gravity. A colloid with Saturn ring defect ($2a = 13$ μm), moving under the action of gravity (tilt angle $\alpha = 12.8°$), moves closer to the well and away from the hill. The period of the wells is decreasing ($\lambda = 70, 60, 50, 40$ μm). As it passes over each successive well, the colloid approaches the structure until the particle finally docks inside the well of most suitable size. The video is 150x faster than real time.
